# Determinants of leptomeningeal collateral flow in stroke patients with a middle cerebral artery occlusion

**DOI:** 10.1007/s00234-016-1727-5

**Published:** 2016-07-20

**Authors:** Tom van Seeters, Geert Jan Biessels, L. Jaap Kappelle, Yolanda van der Graaf, Birgitta K. Velthuis, on behalf of the Dutch acute stroke study (DUST) investigators

**Affiliations:** 1 Department of Radiology, University Medical Center Utrecht, Heidelberglaan 100, HP E01 132, 3584 CX Utrecht, The Netherlands; 2 Department of Neurology, Brain Center Rudolf Magnus, University Medical Center Utrecht, Utrecht, The Netherlands; 3 Julius Center for Health Sciences and Primary Care, University Medical Center Utrecht, Utrecht, The Netherlands

**Keywords:** Leptomeningeal collateral flow, Ischemic stroke, Stroke etiology, Clinical outcome, CT angiography

## Abstract

**Introduction:**

Poor leptomeningeal collateral flow is related to worse clinical outcome in acute ischemic stroke, but the factors that determine leptomeningeal collateral patency are largely unknown. We explored the determinants of leptomeningeal collateral flow and assessed their effect on the relation between leptomeningeal collateral flow and clinical outcome.

**Methods:**

We included 484 patients from the Dutch acute stroke study (DUST) with a middle cerebral artery (MCA) occlusion. The determinants of poor leptomeningeal collateral flow (≤50 % collateral filling) were identified with logistic regression. We calculated the relative risk (RR) of poor leptomeningeal collateral flow in relation to poor clinical outcome (90-day modified Rankin Scale 3–6) using Poisson regression and assessed whether the determinants of leptomeningeal collateral flow affected this relation.

**Results:**

Leptomeningeal collateral flow was poor in 142 patients (29 %). In multivariable analyses, higher admission glucose level (odds ratio (OR) 1.1 per mmol/L increase (95 % CI 1.0–1.2)), a proximal MCA occlusion (OR 1.9 (95 % CI 1.3–3.0)), and an incomplete posterior circle of Willis (OR 1.7 (95 % CI 1.1–2.6)) were independently related to poor leptomeningeal collateral flow. Poor leptomeningeal collateral flow was related to poor clinical outcome (unadjusted RR 1.7 (95 % CI 1.4–2.0)), and this relation was not affected by the determinants of leptomeningeal collateral flow.

**Conclusion:**

Our study shows that admission glucose level, a proximal MCA occlusion, and an incomplete ipsilateral posterior circle of Willis are determinants of leptomeningeal collateral flow that represent a combination of congenital, acquired, and acute factors. After adjustment for these determinants, leptomeningeal collateral flow remains related to clinical outcome.

## Introduction

Leptomeningeal collaterals are a network of small blood vessels connecting distal regions of the intracerebral arterial system [1, 2]. They can provide an alternative route for blood flow to the brain in case of a disruption of the primary blood flow and may therefore protect brain tissue against irreversible damage in case of acute ischemia. Presence of good leptomeningeal collateral flow has been associated with better functional outcomes and smaller infarct volumes after acute ischemic stroke [3–20]. However, factors that determine variation in leptomeningeal collateral flow between patients are largely unknown. Previous studies suggested genetic factors [21] and the vascular risk profile, such as prior hypertension [12, 22, 23], age [22–24], statin use [24], metabolic syndrome [22], and circle of Willis completeness [2], to be related to the patency of leptomeningeal collaterals, but some findings of these studies are contradictory. The presence of an internal carotid artery stenosis or occlusion might also be related to the amount of leptomeningeal collateral flow. In addition, factors that occur at the time of stroke, including higher systolic blood pressure at admission [12], higher blood glucose levels [22], and the location of the occlusion [4], may determine leptomeningeal collateral patency.

In the last decade, CT angiography (CTA) has been successfully incorporated into acute stroke imaging protocols in many stroke centers worldwide and recently has been proven useful to select patients for intra-arterial treatment [25–29]. CTA also offers the possibility to assess the leptomeningeal collateral flow, the circle of Willis, and the extracranial cervical vessels [30]. Leptomeningeal collaterals themselves cannot be visualized directly on CTA, but their patency can be determined by assessing the amount of vascular enhancement in the affected brain area distal of the occluded artery [4, 8]. This is referred to as leptomeningeal collateral flow.

In the present study, we explored possible determinants of leptomeningeal collateral flow in a large prospective cohort of stroke patients with an occlusion of the middle cerebral artery (MCA). We investigated the relation between leptomeningeal collateral flow and clinical outcome and assessed whether this relation was affected by the determinants of leptomeningeal collateral flow.

## Methods

### Study population

All patients participated in the Dutch acute stroke study (DUST), a prospective observational cohort study in six university and eight non-university hospitals in The Netherlands [19]. A detailed description of the DUST study protocol has been published previously [31]. The DUST study population consists of patients with symptoms of acute ischemic stroke of less than 9-h duration, who were enrolled between May 2009 and August 2013. Patients with another diagnosis on admission non-contrast CT (NCCT) such as intracranial hemorrhage were excluded. All patients underwent NCCT, CTA, and CT perfusion (CTP) on admission. Ethical approval was obtained from the medical ethics committee of the University Medical Center Utrecht and local approval from all participating centers. Informed consent was obtained from patients or their legal representatives. The medical ethics committee waived the need for informed consent for patients who died before informed consent could be obtained.

**Fig. 1:**
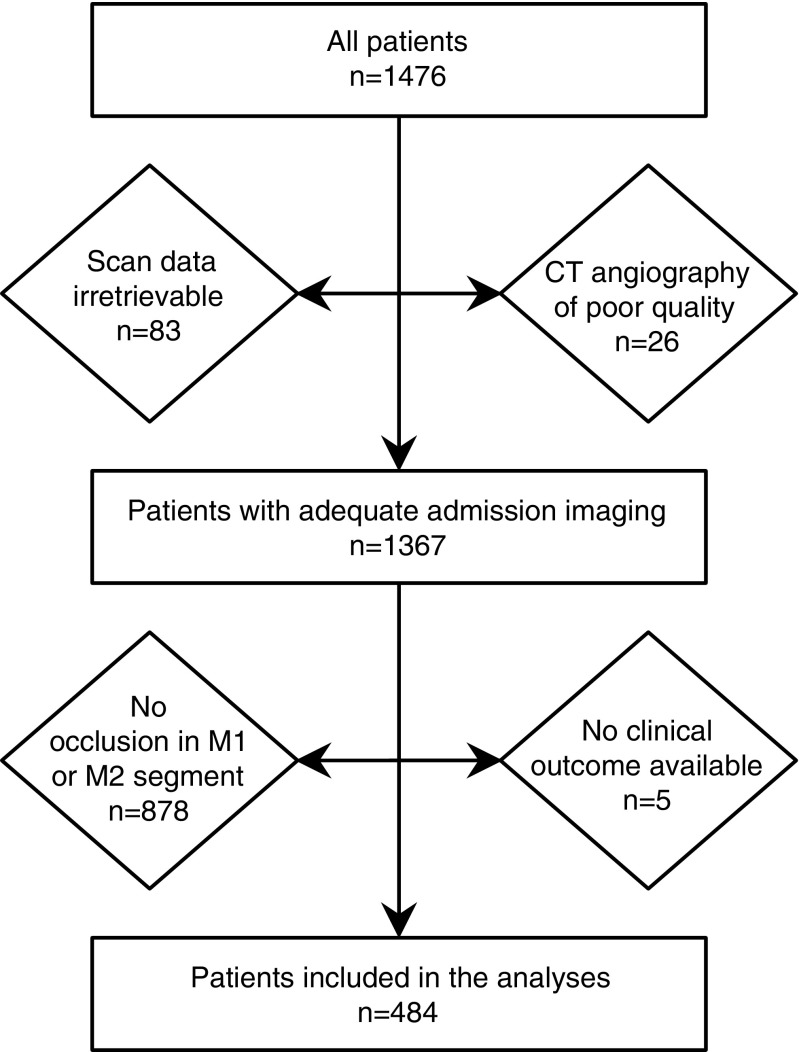
Flowchart depicting the number of patients included in the study

For the present study, we selected patients with a confirmed occlusion in the M1 or M2 segment of the MCA on admission CTA, as leptomeningeal collaterals are needed most if the main blood supply to the downstream territory is completely interrupted. Patients with an isolated occlusion of the intracranial internal carotid artery without an occlusion of the M1 segment were therefore not included (*n* = 42). Patients were excluded if information about clinical outcome after 90 days was missing (Fig. 1).

### Admission CT angiography

All imaging data were assessed by one of three observers with at least 5 years of experience in neurovascular imaging, blinded for all clinical information except for the side of symptoms. A detailed description of the imaging protocol has been described elsewhere [31]. The CTA was scanned from the aortic arch to cranium vertex, and 50–70 mL of contrast material was injected followed by 40 mL of saline with a flow of 6 mL/s. The scan delay after contrast injection was calculated either from time to peak arterial enhancement on CTP, or by a trigger based on Hounsfield unit threshold measurement in the aortic arch.

### Assessment of leptomeningeal collateral flow

**Fig. 2:**
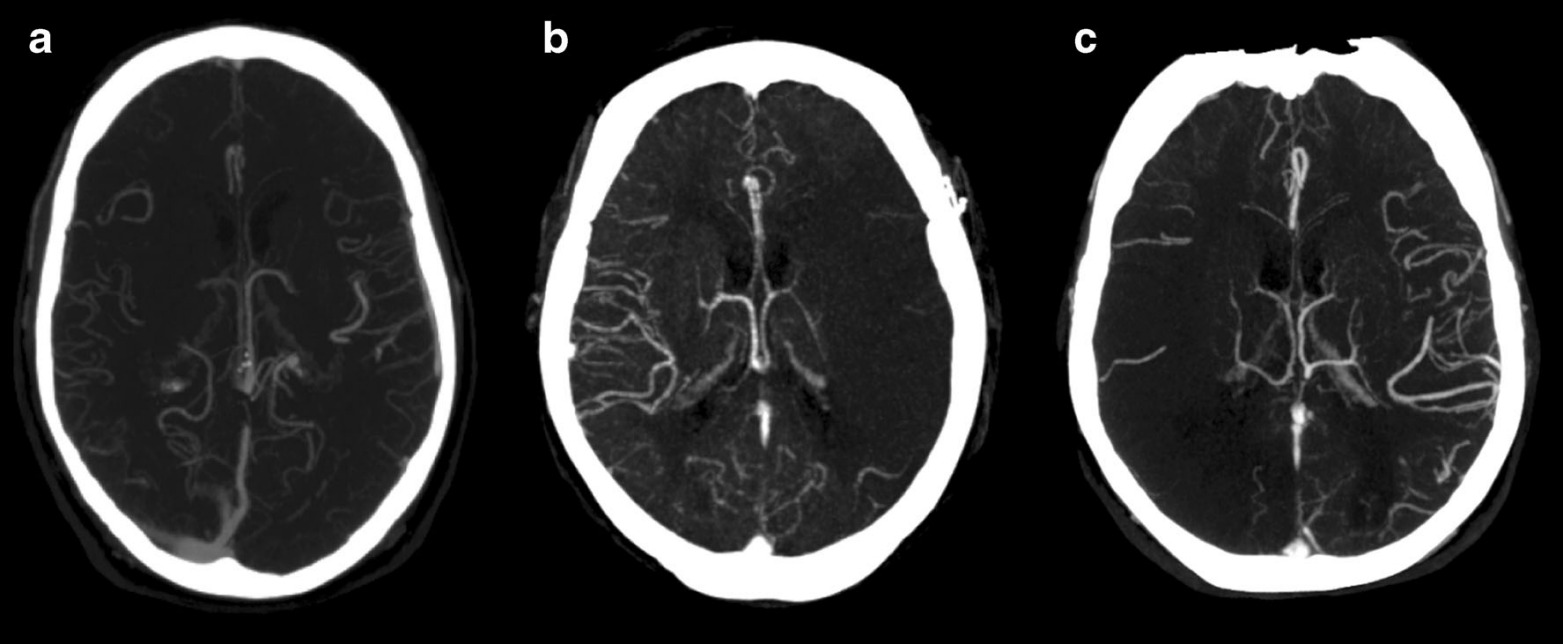
Examples of patients with good (*A*) and poor leptomeningeal collateral flow (*B* and *C*), depicted with 20-mm-thick maximum intensity projection (MIP) reconstructions. *A* Good leptomeningeal collateral flow in a patient with a right-sided ischemic stroke. There was no proximal occlusion and the circle of Willis was complete. The 90-day modified Rankin Scale (mRS) score was 1. *B* Poor leptomeningeal collateral flow in a patient with a left-sided ischemic stroke and a proximal occlusion in the M1 segment. The 90-day mRS was 6. *C* Poor leptomeningeal collateral flow in a patient with a right-sided ischemic stroke and an ipsilateral incomplete posterior circle of Willis. The 90-day mRS was also 6

To assess the leptomeningeal collateral circulation, the observers assessed the CTA source images and maximum intensity projections (MIP) using an interactive sliding slab technique, allowing the observers to increase and decrease the MIP slice thickness (Fig. 2). CTA source images and MIP images were assessed for leptomeningeal collateral flow across the entire MCA territory. The patency of the leptomeningeal collateral circulation was defined as either poor or good [4, 8]. Poor leptomeningeal collateral status was defined as ≤50 % collateral filling of the perfusion territory of the affected MCA or MCA branch territory [4, 8].

### Possible determinants of leptomeningeal collateral flow

#### Patient characteristics

The selection of patient characteristics that were considered potential determinants of leptomeningeal collateral flow was based on previous literature. These determinants included age, prior hypertension, prior diabetes, and systolic blood pressure and glucose level at admission [12, 22–24].

#### Circle of Willis patency

Circle of Willis completeness was assessed on admission CTA. The anterior circle of Willis pathway was considered incomplete if the anterior communicating artery was not visible or if the A1 segment of one of the anterior cerebral arteries was hypoplastic (<1.0 mm), absent, or both. Posterior circle of Willis collaterals were considered incomplete if the posterior communicating artery or P1 segment of the posterior cerebral artery was hypoplastic (<1.0 mm) or absent ipsilateral to the side of the perfusion deficit.

#### Location of the occlusion

The location of the occlusion was also assessed on admission CTA and was classified according the most proximal part of the occlusion, either in the intracranial internal carotid artery, M1 segment, or M2 segment. A proximal MCA occlusion was defined as an occlusion of the M1 segment (with or without an occlusion of the intracranial internal carotid artery).

#### Ipsilateral extracranial internal carotid artery stenosis or occlusion

The ipsilateral extracranial internal carotid artery was assessed on CTA. A stenosis was measured according to the North American Symptomatic Carotid Endarterectomy Trial (NASCET) criteria and was considered significant if it was >70 % [32].

### Clinical outcome

The clinical outcome was assessed after 90 days with the modified Rankin Scale (mRS) [33]. Poor clinical outcome was defined as a score of 3–6, whereas good clinical outcome was defined as a score of 0–2. The mRS was collected by a trained research nurse or neurologist by telephone interview [34].

### Analyses

Single imputation was performed to account for missing data. Continuous variables were truncated at the first and 99th percentile to minimize the effect of outliers [35]. First, we used logistic regression to investigate whether patient characteristics, presence of a proximal MCA occlusion, circle of Willis completeness, or presence of an extracranial >70 % internal carotid artery stenosis or occlusion were related to poor leptomeningeal collateral flow. Multivariable analyses were performed including the significant (*p* < 0.05) variables from the univariable analyses. We did not include stroke severity and the extent of the ischemic area on NCCT and CTA in these analyses, as these are the consequence of poor leptomeningeal collateral flow instead of potential determinants. We also did not adjust for treatment because this cannot be a determinant of leptomeningeal collateral flow, since this treatment is administered after the CTA has been made.

Next, we calculated the relative risk (RR) for the relation between leptomeningeal collateral flow and clinical outcome using Poisson regression [36]. We investigated whether this relation was affected by the factors that were associated with leptomeningeal collateral flow, by evaluating the change of the RR in multivariable analyses after adjusting for these factors. We also assessed possible interaction effects between these factors and patency of leptomeningeal collateral flow in relation to clinical outcome. As stroke severity and treatment can be confounding factors for the relation between leptomeningeal collateral flow and clinical outcome, we included them in the Poisson regressions. All analyses were performed with R version 3.0.2.

## Results

A total of 489 patients had an occlusion of an M1 or M2 segment on admission CTA (Fig. 1). After exclusion of 5 patients (1.0 %) with missing information on clinical outcome, 484 patients remained for the analyses. The mean age was 66.6±14.6 years, 268 (55 %) patients were male, and the median admission NIHSS was 13 (interquartile range 8–17). The most proximal part of the occlusion was located in the M1 segment in 234 patients (48 %) and in the M2 segment in 183 patients (38 %). In the remaining 67 patients (14 %), there was an occlusion of the intracranial internal carotid artery in combination with an occlusion of the M1 segment. Leptomeningeal collateral flow was poor in 142 patients (29 %) and good in 342 patients (71 %). At 90 days, 251 patients (52 %) had a poor clinical outcome. Additional patient characteristics can be found in Table 1.

**Table 1:** Patient characteristics (*n* = 484)

Age (years)	66.6 (14.6)
Male gender	268 (55.4)
Stroke severity (NIHSS)	13 (8–17)
Time from symptom onset to scan (minutes)	100 (64–159)
Treatment (IV-rtPA, IAT, or mechanical thrombectomy)	361 (74.6)
Smoking	144 (29.8)
Glucose (mmol/L)	6.7 (5.9–7.8)
Systolic blood pressure (mmHg)	151 (26.5)
Diastolic blood pressure (mmHg)	84 (17.2)
Medical history	
Ischemic stroke or transient ischemic attack	87 (18.0)
Hypertension	249 (51.4)
Diabetes	61 (12.6)
Hyperlipidemia	137 (28.3)
Atrial fibrillation	79 (16.3)
CT angiography findings	
Poor leptomeningeal collateral flow	142 (29.3)
Location of intracranial occlusion	
Intracranial internal carotid artery (and M1 segment)	67 (13.8)
M1 segment (without intracranial internal carotid artery)	234 (48.3)
M2 segment	183 (37.8)
Circle of Willis	
Complete anterior and ipsilateral posterior CoW	152 (31.4)
Incomplete ipsilateral posterior CoW, complete anterior CoW	296 (61.2)
Incomplete anterior CoW, complete ipsilateral posterior CoW	8 (1.7)
Incomplete anterior and ipsilateral posterior CoW	28 (5.8)
Significant ipsilateral extracranial >70 % internal carotid artery stenosis or occlusion	133 (27.5)

All data are displayed as mean (standard deviation), median (interquartile range), or *n* (%)

*NIHSS* National Institutes of Health Stroke Scale, *IV-rtPA* intravenous thrombolysis with recombinant tissue type plasminogen activator, *IAT* intra-arterial thrombolysis, *CoW* circle of Willis

### Determinants of leptomeningeal collateral flow

Univariable logistic regression analyses showed that higher admission glucose levels, presence of a proximal intracranial occlusion, and an incomplete ipsilateral posterior circle of Willis were related to poor leptomeningeal collateral flow (Table 2). Age, systolic blood pressure at admission, prior hypertension, prior diabetes, and presence of a significant internal carotid artery stenosis or occlusion were not related to poor leptomeningeal collateral flow.

**Table 2:** Univariable analyses for the association of patient characteristics and imaging findings with leptomeningeal collateral flow (*n* = 484)

	Leptomeningeal collateral flow			
	Good (*n* = 342)	Poor (*n* = 142)	Odds ratio	95 % confidence interval
Patient characteristics				
Age (years, odds ratio per decade)	66.2 (14.6)	67.5 (14.6)	1.06	0.93–1.21
Systolic blood pressure (mmHg, odds ratio per 10 mmHg)	152 (26.4)	150 (27.0)	0.97	0.90–1.04
Glucose (mmol/L, odds ratio per mmol/L)	6.6 (5.8–7.7)	6.7 (6.2–8.4)	1.10	1.01–1.20
Hypertension	174 (50.9)	75 (52.8)	1.08	0.73–1.60
Diabetes	43 (12.6)	18 (12.7)	1.01	0.56–1.82
Hyperlipidemia	99 (28.9)	38 (26.8)	0.90	0.58–1.39
CT angiography findings				
Proximal MCA occlusion^a^	198 (57.9)	103 (72.5)	1.92	1.25–2.94
Circle of Willis				
Complete anterior and ipsilateral posterior CoW	116 (33.9)	36 (25.4)	Reference	Reference
Incomplete ipsilateral posterior CoW, complete anterior CoW	197 (57.6)	99 (69.7)	1.62	1.04–2.53
Incomplete anterior CoW, complete ipsilateral posterior CoW	7 (2.0)	1 (0.7)	0.46	0.05–3.87
Incomplete anterior and ipsilateral posterior CoW	22 (6.4)	6 (4.2)	0.88	0.33–2.33
Significant ipsilateral extracranial >70 % internal carotid artery stenosis or occlusion	93 (27.2)	40 (28.2)	1.05	0.68–1.62

Data are displayed as mean (standard deviation), median (interquartile range), or *n* (%)

*MCA* middle cerebral artery, *CoW* circle of Willis

^a^Defined as an occlusion of the M1 segment with or without an occlusion of the intracranial internal carotid artery

All variables that were determinants of poor leptomeningeal collateral flow in univariable analyses were also related to poor leptomeningeal collateral flow in multivariable logistic regression analyses (Table 3). These analyses show that higher admission glucose levels (OR 1.1 per mmol/L increase (95 % CI 1.0–1.2)), presence of a proximal MCA occlusion (OR 1.9 (95 % CI 1.3–3.0)), and an incomplete posterior circle of Willis (OR 1.7 (95 % CI 1.1–2.6)) were independently related to poor leptomeningeal collateral flow.

**Table 3:** Multivariable analyses for the association of patient characteristics and imaging findings with poor leptomeningeal collateral flow (*n* = 484)

	Odds ratio	95 % confidence interval
Glucose (per mmol/L)	1.10	1.01–1.20
Proximal MCA occlusion^a^	1.94	1.26–2.99
Circle of Willis		
Complete anterior and ipsilateral posterior CoW	Reference	Reference
Incomplete ipsilateral posterior CoW, complete anterior CoW	1.66	1.06–2.61
Incomplete anterior CoW, complete ipsilateral posterior CoW	0.37	0.04–3.15
Incomplete anterior and ipsilateral posterior CoW	1.02	0.37–2.76

*MCA* middle cerebral artery, *CoW* circle of Willis

^a^Defined as an occlusion of the M1 segment with or without an occlusion of the intracranial internal carotid artery

### Determinants of leptomeningeal collateral flow: effect on the relation with clinical outcome

Poor clinical outcome was more frequent in patients with poor leptomeningeal collateral flow (103/142 patients, 73 %) than in patients with good leptomeningeal collateral flow (148/342 patients, 43 %). The corresponding unadjusted RR for the relation between poor leptomeningeal collateral flow and poor clinical outcome was 1.7 (95 % CI 1.4–2.0). In multivariable analyses this relation was not affected by the determinants of poor leptomeningeal collateral flow: admission glucose level, presence of a proximal MCA occlusion, and circle of Willis completeness (Table 4). Also, after additionally adjusting for other confounders (stroke severity and treatment), the relation between poor leptomeningeal collateral flow and poor clinical outcome was not affected. Furthermore, there was no interaction of admission glucose level, presence of a proximal MCA occlusion, and circle of Willis completeness with the patency of leptomeningeal collaterals for their relation with clinical outcome (all interaction term *p* values >0.05).

**Table 4:** Relative risks for the relation between poor leptomeningeal collateral flow and poor clinical outcome (*n* = 484)

	Relative risk	95 % confidence interval
Unadjusted	1.68	1.43–1.96
Adjusted for		
Glucose	1.63	1.39–1.91
Proximal MCA occlusion^a^	1.54	1.32–1.80
Additionally adjusted for stroke severity (NIHSS)	1.31	1.12–1.53
Additionally adjusted for treatment (IV-rtPA, IAT, or mechanical thrombectomy)	1.55	1.33–1.80
Circle of Willis completeness	1.66	1.41–1.94

*MCA* middle cerebral artery, *NIHSS* National Institutes of Health Stroke Scale, *IV-rtPA* intravenous thrombolysis with recombinant tissue type plasminogen activator, *IAT* intra-arterial thrombolysis

^a^Defined as an occlusion of the M1 segment with or without an occlusion of the intracranial internal carotid artery

## Discussion

Our study shows that higher admission glucose level, the presence of an occlusion of the proximal MCA, and the presence of an incomplete posterior circle of Willis were determinants of poor leptomeningeal collateral flow in acute stroke patients. Poor leptomeningeal collateral flow was related to worse clinical outcome, but this relation was not influenced by admission glucose level, by the presence of a proximal MCA occlusion, or by the completeness of the circle of Willis.

Different factors may determine the patency of leptomeningeal collaterals in patients with an acute ischemic stroke. In mice, differences in genetic background are related to the extent of leptomeningeal collateral flow [21], and this could also play a role in humans, possibly due to innate inter-individual differences in the cerebral arterial vascular tree. Furthermore, we showed that an incomplete posterior circle of Willis ipsilateral to the occlusion, in combination with a complete anterior circle of Willis, was related to poor leptomeningeal collateral flow. This finding may be explained partly by the lack of leptomeningeal collaterals between the posterior cerebral artery and MCA territories in patients with a fetal type posterior circle of Willis [2]. Furthermore, patency of circle of Willis collaterals might be an expression of the potential to recruit collateral systems, possibly due to the same underlying genetic mechanisms as for the development of leptomeningeal collaterals [21]. Other circle of Willis variants that were investigated were not related to leptomeningeal collateral flow, although the numbers of patients with the combination of an incomplete anterior circle of Willis and a complete posterior circle of Willis (*n* = 8) and patients with an incomplete anterior and incomplete posterior circle of Willis (*n* = 28) are too low for firm conclusions.

Other acquired factors that potentially could determine the patency of leptomeningeal collaterals include vascular risk factors and the presence of a significant stenosis or occlusion of the internal carotid artery. The latter may also affect the size of the circle of Willis collaterals. Previous studies about associations of vascular risk factors with leptomeningeal collaterals were inconclusive. These studies suffered from problems such as small sample sizes [23, 24], retrospective study designs [23, 24], or rather simple comparative analyses instead of dedicated (regression) analyses to assess the relation with leptomeningeal collaterals [12, 22, 23]. In our large prospective study, we did not find a relation between age, hypertension, diabetes, and hyperlipidemia and poor leptomeningeal collateral flow. Neither did we find a relation between the presence of a >70 % stenosis or occlusion of the internal carotid artery and the patency of leptomeningeal collaterals. Therefore, our results suggest that exposure to vascular risk factors for atherosclerosis is not a major contributor to the recruitment of leptomeningeal collaterals in acute ischemic stroke. This is in contrast to the collaterals that are recruited in response to chronic hypoxemia in peripheral artery disease [37]. However, further studies including other vascular risk factors (e.g., diet, weight, physical activity, cholesterol) and other signs/markers of existing atherosclerosis should be performed to further clarify the relation between cardiovascular risk factors and leptomeningeal collateral flow.

Acute factors that are present at the time of stroke and that may determine the patency of leptomeningeal collaterals include admission systolic blood pressure, admission glucose level, and the location of the occlusion. Higher systolic blood pressure at admission was not related to poor leptomeningeal collateral flow in our study. One other study has shown that patients with increased leptomeningeal collateral flow have a lower systolic blood pressure than patients with normal or decreased leptomeningeal collateral flow [12], but no other study has confirmed this. Admission hyperglycemia has been related previously to both poor clinical outcome after ischemic stroke [38] and to poor leptomeningeal collateral flow [22]. Our results also show a relation between a higher admission glucose level and poor leptomeningeal collateral flow, but it remains unclear whether this is a true causal relation. Animal studies have demonstrated that acute hyperglycemia could lead to endothelial dysfunction and result in impaired collateral function [39]. Stress hyperglycemia in patients with acute ischemic stroke has been associated with poor leptomeningeal collateral flow [22]. Furthermore, acute hyperglycemia can reflect a pre-existing but unrecognized diabetes. Data on HbA1c, indicating long-term elevated glucose levels, may be used to differentiate between stress hyperglycemia and diabetes [38], but could not be analyzed as this was missing in 36 % of the patients in our study. However, as we did not find a relation between prior diabetes and poor leptomeningeal collateral flow, the effect of stress hyperglycemia probably dominates the effect of pre-existing diabetes.

The observation that patients with a proximal MCA occlusion often have poor leptomeningeal collateral flow has been shown in recent studies [4, 12, 40]. In our study, the proportion of patients with poor leptomeningeal collateral flow was 61 % higher if there was an occlusion of the M1 segment, instead of a more distal occlusion. There may be several explanations for this finding. First, small ischemic areas in patients with a distal occlusion are surrounded on all sides by adequately perfused brain tissue, including areas supplied by non-occluded M2 branches, allowing for better leptomeningeal collateral flow. On the other hand, patients with a proximal MCA occlusion have larger ischemic areas, less surrounding adequately perfused brain tissue, and hence there are less opportunities for sufficient leptomeningeal collateral flow. Another explanation could be that contrast filling of distal branches via leptomeningeal collaterals has not yet occurred in patients with a proximal MCA occlusion at the time the CTA is performed, as the time needed for contrast arrival via leptomeningeal collaterals might be longer for patients with an M1 occlusion than for patients with a more distal occlusion. This means that our study could have overestimated the number of patients with a proximal occlusion that have poor leptomeningeal collateral flow.

CTA is often used in clinical practice to image the cerebral vasculature in acute ischemic stroke and is increasingly important to select patients for endovascular treatment. As such, it is important to investigate leptomeningeal collateral flow as seen on CTA. Newer techniques such as CTA reconstruction from CTP data and multiphase CTA make the CTA independent from timing of contrast administration and scan acquisition and could improve assessment of the collateral circulation [41, 42]. However, these techniques are currently not widely available for use in clinical practice. Furthermore, the thin slice CTP data that is needed for high-quality reconstruction of CTA from CTP was missing in a substantial part of our cohort. We felt that it was more important to have a large sample size with enough power for our analyses than to compromise on this when using newer CTA from CTP techniques. The above considerations made us decide to use standard CTA instead of newer CTA techniques. Regarding the location of the occlusion, we classified all occlusions to the most proximal part of the occlusion. A limitation is that this does not take the length of the thrombus into account. Furthermore, we used a dichotomized classification of leptomeningeal collateral flow in our analyses. Although previous studies also used this approach [8, 18, 43], this may oversimplify the complex pathophysiology of stroke hemodynamics. Nonetheless, through the use of a dichotomized score, the analyses could be performed with logistic regression, making the results better understandable and interpretable for many physicians.

## Conclusions

Our study shows that admission glucose level, a proximal MCA occlusion, and an incomplete ipsilateral posterior circle of Willis are determinants of leptomeningeal collateral flow that represent a combination of congenital, acquired, and acute factors. After adjustment for these determinants, leptomeningeal collateral flow remains related to clinical outcome.
